# Advances in mixed-matrix membranes for biorefining of biogas from anaerobic digestion

**DOI:** 10.3389/fchem.2024.1393696

**Published:** 2024-06-03

**Authors:** Jean Carlo Guerrero Piña, Daniel Alpízar, Paola Murillo, Mónica Carpio-Chaves, Reynaldo Pereira-Reyes, José Vega-Baudrit, Claudia Villarreal

**Affiliations:** ^1^ Escuela de Ciencia e Ingeniería de Materiales, Instituto Tecnológico de Costa Rica, Cartago, Costa Rica; ^2^ Laboratorio Nacional de Nanotecnología (LANOTEC), Centro Nacional de Alta Tecnología (CENAT), San José, Costa Rica; ^3^ Escuela de Ingeniería en Seguridad Laboral e Higiene Ambiental, Instituto Tecnológico de Costa Rica, Cartago, Costa Rica

**Keywords:** biogas, biorefining, gas separation, polymeric membrane, mixed-matrix membrane, biomethane, nanofiller

## Abstract

This article provides a comprehensive review of the state-of-the-art technology of polymeric mixed-matrix membranes for CO_2_/CH_4_ separation that can be applied in medium, small, and domestic biogas systems operating at low pressures (0.2–6 kPa). Critical data from the latest publications of CO_2_/CH_4_ separation membranes were analyzed, considering the ratio of CO_2_/CH_4_ permeabilities, the CO_2_ selectivity, the operating pressures at which the membranes were tested, the chemistry of the polymers studied and their gas separation mechanisms. And the different nanomaterials as fillers. The intrinsic microporous polymers (PIMs) were identified as potential candidates for biomethane purification due to their high permeability and selectivity, which are compatible with operation pressures below 1 bar, and as low as 0.2 bar. This scenario contrasts with other polymers that require pressures above 1 bar for operation, with some reaching 20 bar. Furthermore, the combination of PIM with GO in MMMs was found to not influence the permeability significantly, but to contribute to the membrane stability over time, by preventing the structural collapse of the membrane caused by aging. The systematic analysis here presented is a valuable resource for defining the future technological development of CO_2_/CH_4_ separation membranes for biogas biorefining.

## 1 Introduction

Biogas is a strategic fuel against the dependence on fossil fuels, especially to replace natural gas (Atelge et al., 2021). Biogas is a mixture of gases with a high content of methane, and it is produced by the anaerobic digestion of biomass, with a composition as detailed in Table 1 (Rick Wanek, 2011; Occupational Safety and Health Administration OSHA, 2019; Piergrossi et al., 2019; Yang et al., 2020). The upgrading of biogas refers to physical, chemical, and biological processes to enhance its properties by removing unwanted compounds and producing purified biomethane. Carbon dioxide (CO_2_) is the second most abundant component in biogas, and it is chemically inert to the combustion reaction. However, CO_2_ reduces the calorific value of the biogas to 20–25 MJ/m³, compared to biomethane with 35–40 MJ/m³. Other undesirable components in biogas include water, hydrogen sulfide (H_2_S), and siloxanes, which even at trace levels can cause corrosion, deteriorate the biogas systems, and potentially harm human health (Baena-Moreno et al., 2019).

**TABLE 1:** Common components of biogas obtained by anaerobic digestion of biomass. Based on [(Occupational Safety and Health Administration OSHA, 2019; Rick Wanek, 2011; Yang et al., 2020; Piergrossi et al., 2019)].

Compounds	Concentration	Adverse effects on biogas
Methane CH_4_	35–85 (vol%)	Global Warning Potential (GWP) of CH_4_ is 21 times greater than CO_2_ if it doesn’t burn
Carbon Dioxide CO_2_, Nitrogen N_2_, Oxygen O_2_, Hydrogen H_2_	10–65 (vol%)	
		Reduces CH_4_ purity and the calorific value of the biogas
Hydrogen Sulfide H_2_S	0–5,000 ppm	Corrosive and toxic
Ammonia NH_3_	0–5 ppm	
Siloxanes C_n_H_m_Si_x_O_y_	0–60 ppm	Forms SiO_2_ that clogs pipes and valves in biogas systems
Water H_2_O	0–1 (vol%)	Promotes corrosion

Millions of biogas plants are currently in operation in the world ranging from domestic to very industrial scales. Anaerobic digestion technology integrates a wide variety of biosystems, in contrast to natural gas refining that currently takes place at a few hundred industrial facilities around the world. Various cleaning methods have been developed to improve the quality of the biogas at small and medium scale, mainly to remove H_2_S, siloxanes, and water. However, there are no widespread or affordable methods for CO_2_ removal for smaller scales (Sanders et al., 2013; Sutanto et al., 2017). Specific polymer membranes and their composites with fillers in mixed-matrix membranes (MMMs) have been shown to selectively separate gas mixtures taking advantage of both the physical and chemical interactions between membrane material and gas molecules (Vu et al., 2003; Cheng et al., 2019). MMM refining has the potential to be incorporated into biogas systems as a scalable and flexible technology for different system dimensions. MMM technology has lower costs, easier operation, reduced equipment size, higher efficacy, and a smaller carbon footprint than other capture and filtration technologies, such as adsorption filtration systems and cryogenic distillation; or chemical methods like caustic soda washing (Chuah et al., 2021). Therefore, the MMM technology for CH_4_/CO_2_ gas separation and biogas upgrading is feasible to implement in systems at smaller scales.

This review presents a comprehensive and updated visualization of the advances in the adaptation of polymeric membrane technology and MMMs for biogas biorefining in medium- and small-scale biogas systems. This review is elaborated by collecting, processing, and analyzing the main characteristics and variables of polymeric membranes used for CH_4_/CO_2_ gas separation and their modification with fillers. The objective is to identify the optimal characteristics for MMMs in the context of medium and small-scale anaerobic digestion, considering the impact these systems would have in practice, such as operating conditions, component separation efficiencies and reported yields, which are the main indicator for development of anaerobic digestion projects. This work contributes to the development of sustainable and efficient solutions for biogas biorefinery as a renewable resource of growing importance in the transition to a low-carbon economy, as an alternative for countries with developing economies.

## 2 Mechanisms for gas separation through polymeric membranes

The permeability is an intrinsic property of a gas in a specific material, reflecting its inherent capacity to allow molecules to go through under specific conditions. Permeability (*P*) is defined in Eqs 1, 2 by the penetrant diffusion coefficient of transport through the membrane (*D*), the solubility coefficient (*S*), the penetrant concentration (*C*), and partial pressure (*p)* (Galizia et al., 2017):

P=D×S (1)

S=C/p (2)

**FIGURE 1:**
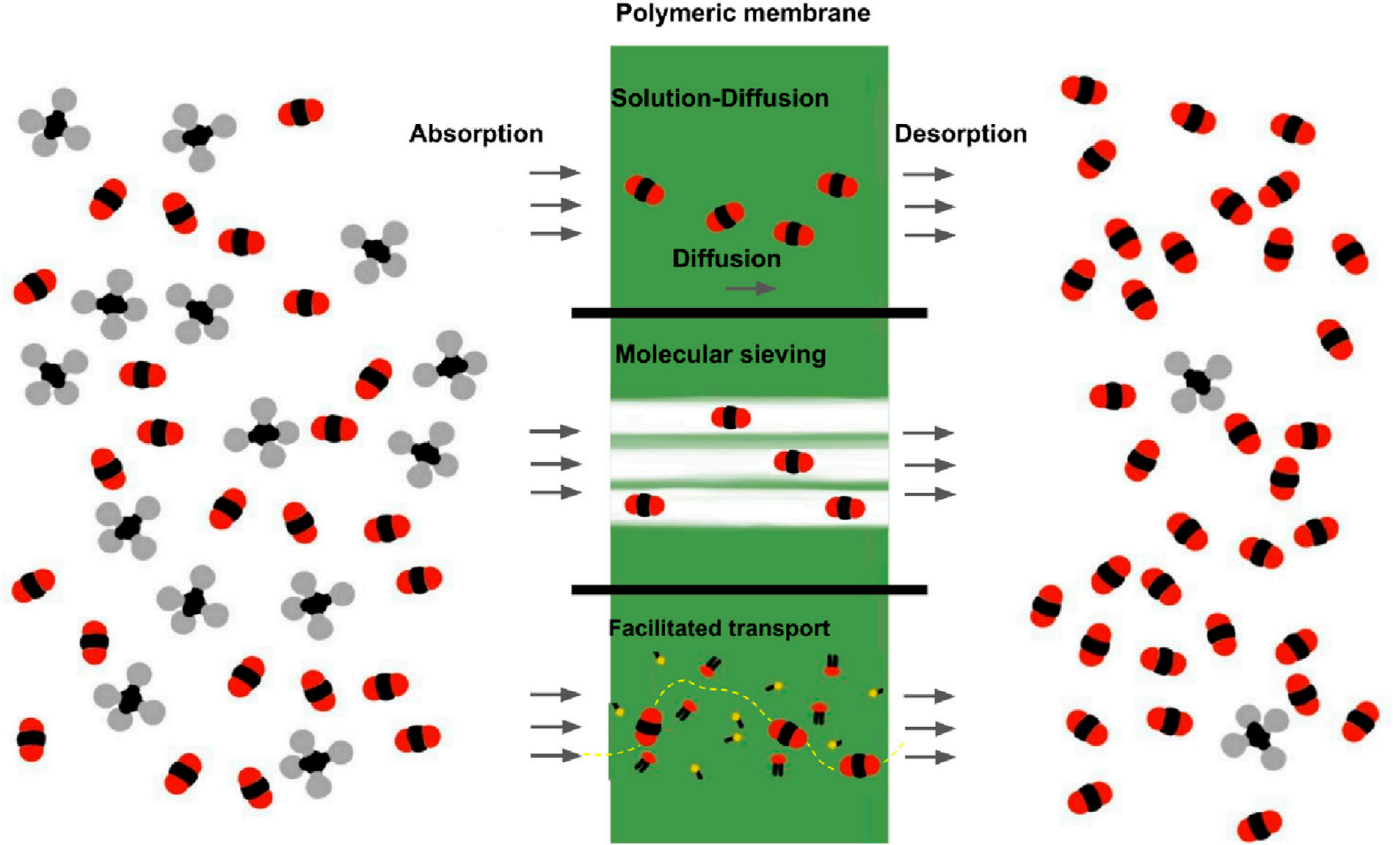
Mechanisms of gas separation in polymeric membranes: solution-diffusion, molecular sieving, and facilitated transport through functional groups. Based on (Li et al., 2015; Cheng et al., 2019).

The permeability and solubility are influenced by the chemical and physical interactions between the permeating molecules and the membrane material. The transport of molecules through membranes relies on diffusion, solution, adsorption, and molecular sieving mechanisms within the polymer matrix as illustrated in Figure 1 (Li et al., 2015; Cheng et al., 2019). The transport process begins with the absorption of gas molecules onto the membrane surface. Then, the diffusion through the membrane matrix is driven by the concentration gradient, the affinity of the gas molecules for the polymer matrix and the ratio of size and shape of the molecules relative to the pore size within the membrane structure. For biogas upgrading into biomethane, the selectivity of either CH_4_ or CO_2_ is achieved by taking advantage of the differences in the physical and chemical interactions of the molecules with the membrane material. The sieving effect allows, by kinetic diameter, selectively pass through the molecules through the membrane pores. Additionally, the chemical affinity between specific gas molecules and the membrane material can enhance selectivity by preferentially absorbing certain species (Li et al., 2015; Cheng et al., 2019).

## 3 Historical development of mixed-matrix membrane technology for gas separation

**FIGURE 2:**
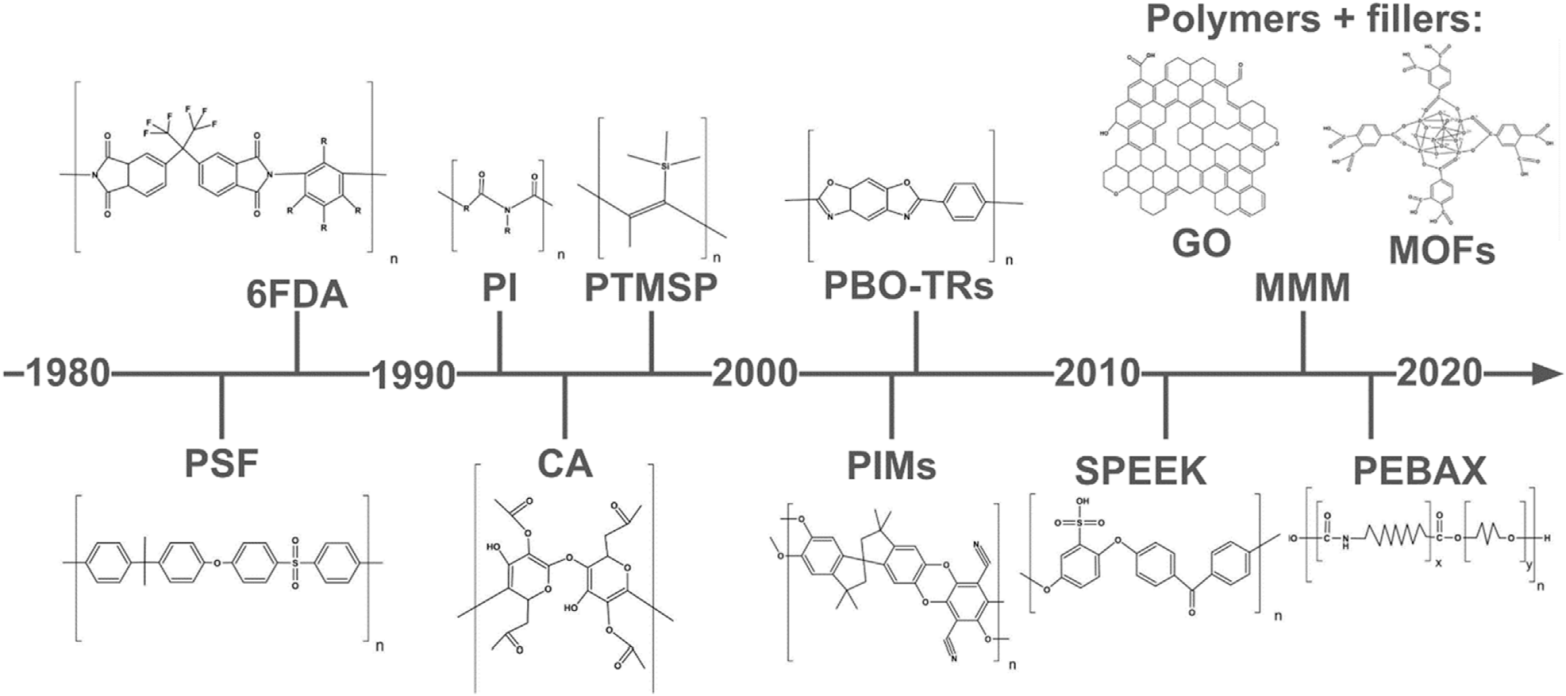
Timeline of innovations in polymeric membranes for gas separation. Based on (Bollinger et al., 1982; Mc Keown and Budd, 2006; Scholes et al., 2012; Sanders et al., 2013; Li et al., 2015; Jansen et al., 2016; Liu et al., 2016; Ali et al., 2017; Galizia et al., 2017).

In 1979, within the context of natural gas refining, the first gas separation station using polymeric membranes made of polysulfone (PSF) was built (Galizia et al., 2017). Commercial PSF membrane technology was developed by Permea, specifically for H_2_, N_2_, Ar and CH_4_ purification plants (Bollinger et al., 1982). The polymer membranes developed between the 1980s and 1990s account for 80%–90% of today’s membrane separation industry (Galizia et al., 2017). Since then, different materials have been investigated and implemented. Figure 2 presents a timeline of the innovations of polymeric membranes from 1980 to date (Bollinger et al., 1982; Mc Keown and Budd, 2006; Scholes et al., 2012; Sanders et al., 2013; Li et al., 2015; Jansen et al., 2016; Liu et al., 2016; Ali et al., 2017; Galizia et al., 2017). H_2_ recovery, N_2_ production, natural gas treatment and vapor recovery applications were estimated at $1.5 billion annually by 2017, with natural gas treatment alone accounting for $300 million per year (Chuah et al., 2021).

Around 1990, different super glassy polymers were developed for CO_2_/CH_4_ separation, such as cellulose acetate (CA), polyphenylene oxide (PPO), and poly (1-trimethylsilyl-1-propyne) (PTMSP) (Park et al., 2008; Koros et al., 1988; Puleo et al., 1989; Sada et al., 1989; Pinnau, 1996; Jansen et al., 2016). Around the 2000s, there were advancements in the performance of the membranes investigated with the introduction of thermally reorganized polymers (TR), which exhibit a microporous character, being polyimides (PI) the excelling TR (Mizumoto et al., 1993; Hachisuka et al., 1995; Al-Masri et al., 1999; Zimmerman and Koros, 1999; Yang et al., 2001; Nagel et al., 2002; Shida et al., 2006; Wang et al., 2007; Liu et al., 2016). Other TR, such as polyphenylene benzobisoxazole (PBO) exhibited outstanding permeability/selectivity ranges, in exchange for a detriment in mechanical performance (Mc Keown and Budd, 2006). Polymers with intrinsic microporosity (PIM) have emerged, in the same selectivity/permeability trade off zone as TR as materials with higher CO_2_ permeability. PIM varieties have a structure made up of cyclic chains, which, when polymerized, form structures with limited packing density, generating high internal void volumes that result in higher permeability. PIM-1 has been one of the most researched in CO_2_/CH_4_ separation (Scholes et al., 2012; Ali et al., 2017).

In recent years, advances in membrane technology have shifted towards the incorporation of fillers into polymer matrices, resulting in mixed-matrix membranes (MMMs). Some of the incorporated fillers are carbonous materials, graphene oxide (GO) and few layer graphene (FLG), metal-organic frameworks (MOFs), and organosilicon materials as polyhedral oligomeric silsesquioxane (POSS), which significantly enhance the longevity of their properties reducing problems as aging and plasticization (Shen et al., 2015; Xin et al., 2015; Castarlenas et al., 2017; Feijani et al., 2018; Al-Maythalony, 2019; Li et al., 2020). While the inclusion of fillers offers promising avenues for enhancing the membrane performance, challenges such as dispersion of the filler within the polymer matrix, the potential for agglomeration, and ensuring the long-term stability and resistance to fouling must be addressed (Li et al., 2015; Cheng et al., 2019).

The polymeric MMMs developed for the separation of CH_4_/CO_2_ mixtures present obstacles for their implementation in low pressure biogas systems since their physical characteristics are not able to obtain viable separations with these operating conditions (Ebrahimi et al., 2016; Kheirtalab et al., 2020; Raouf et al., 2020; Sainath et al., 2021). Their performance over time, in which the physical aging and plasticization of the polymer reduces the permeability and selectivity is also a concern that limits their application to industrial scales. Although several polymers have shown outstanding properties so far, the natural gas refining industry is still largely dominated by CA, with 80% of the 2012 market dominated, followed by PI (Sanders et al., 2013; Jansen et al., 2016).

More recently, with the development of nanomaterials for implementation in MMM, ultrathin membranes from combinations of nanofillers have also been developed, with exceptional results in their performance. Researchers have developed graphene oxide membranes with ZIF-8, organosilica with reduced graphene oxide, and individual sheets of graphene oxide, among others. Despite their great performance, this type of membrane lacks two-of-three fundamental aspects in the list required for its implementation: performance-feasibility of implementation-manufacturing costs (Li et al., 2018; Sainath et al., 2021; Zhao et al., 2021).

### 3.1 Analysis of mixed-matrix membranes performance for biomethane purification

The implementation of membranes for biomethane purification at small and medium scale anaerobic digestion systems requires their viable operation at low pressure. The data of separation performance of membranes for CH_4_/CO_2_ separation from four previous major reviews (Koros et al., 1988; Vu et al., 2003; Galizia et al., 2017; Comesaña-Gándara et al., 2019), and multiple investigations (Park et al., 2008; Koros et al., 1988; Puleo et al., 1989; Sada et al., 1989; Mizumoto et al., 1993; Hachisuka et al., 1995; Pinnau, 1996; Al-Masri et al., 1999; Zimmerman and Koros, 1999; Lin and Chung, 2001; Yang et al., 2001; Nagel et al., 2002; BUDD et al., 2005; Shida et al., 2006; Wang et al., 2007; Ghanem et al., 2009; Fritsch et al., 2011; Ma et al., 2012; Li et al., 2015; Shen et al., 2015; Xin et al., 2015; Althumayri et al., 2016; Ebrahimi et al., 2016; Bernardo et al., 2017; Castarlenas et al., 2017; Alberto et al., 2018; Feijani et al., 2018; Li et al., 2018; Al-Maythalony, 2019; Kheirtalab et al., 2020; Li et al., 2020; Raouf et al., 2020; Yang et al., 2020; Luque-Alled et al., 2021a; Sainath et al., 2021; Zhao et al., 2021; Mohsenpour et al., 2022) have been analyzed and visually organized in this section (See database in Supplementary Material). The data is presented in graphs of CO_2_/CH_4_ selectivity versus CO_2_ permeability and incorporate the visualization of variables such as polymer, fillers, and the year of publication of the research. The pressure at which the gas separation was carried out is also included in the analysis, which is a variable that may not be as important for large industrial natural gas operations but is very significant for low-pressure biogas systems. Special attention has been devoted to recent research on MMMs using different fillers. The Pineau (2015) and Robeson (2008) describe limits based of permeability vs. selectivity historic data points, in order to describe the behavior of Limits that were added to the graphs as benchmarks, derived from other studies and described in equations (3–5) (Comesaña-Gándara et al., 2019), where $\alpha_{{CO}_{2}/{CH}_{4}}$ is the separation factor of the gases.

$$\text{Pineau Limit (2015)}\;P_{CO2}=22.584\times 10^{6}\alpha_{CO2/CH4}^{-2.401}$$ (3)

$$\text{Robeson Limit (2008)}\;P_{CO2}=5.369\times 10^{6}\alpha_{CO2/CH4}^{-2.636}$$ (4)

$$\alpha_{{CO}_{2}/{CH}_{4}}=P_{CO2}/P_{{CH}_{4}}$$ (5)

**FIGURE 3:**
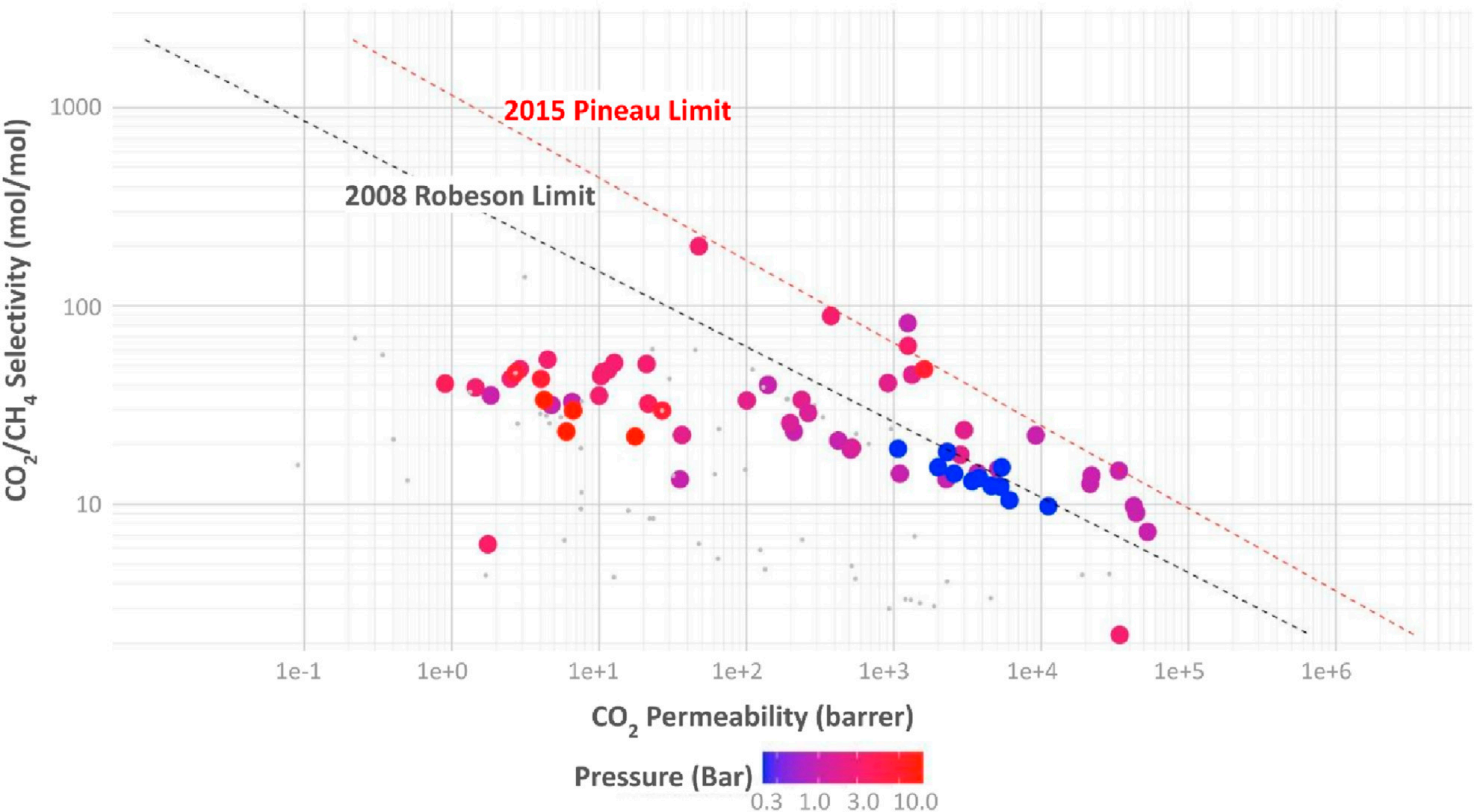
CO_2_/CH_4_ selectivity vs. CO_2_ permeability for different polymeric MMMs in gas separation, indicating the pressure used for each measurement in the color scale. Based on (Park et al., 2008; Koros et al., 1988; Puleo et al., 1989; Sada et al., 1989; Mizumoto et al., 1993; Hachisuka et al., 1995; Pinnau, 1996; Al-Masri et al., 1999; Zimmerman and Koros, 1999; Lin and Chung, 2001; Yang et al., 2001; Nagel et al., 2002; Vu et al., 2003; BUDD et al., 2005; Shida et al., 2006; Wang et al., 2007; Ghanem et al., 2009; Fritsch et al., 2011; Ma et al., 2012; Sanders et al., 2013; Li et al., 2015; Shen et al., 2015; Xin et al., 2015; Althumayri et al., 2016; Ebrahimi et al., 2016; Bernardo et al., 2017; Castarlenas et al., 2017; Galizia et al., 2017; Alberto et al., 2018; Feijani et al., 2018; Li et al., 2018; Al-Maythalony, 2019; Comesaña-Gándara et al., 2019; Kheirtalab et al., 2020; Li et al., 2020; Raouf et al., 2020; Yang et al., 2020; Luque-Alled et al., 2021a; Luque-Alled et al., 2021b; Sainath et al., 2021; Zhao et al., 2021; Mohsenpour et al., 2022).

Figure 3 shows the selectivity of CO_2_/CH_4_ as a function of CO_2_ permeability, and the pressure of operation is indicated as a color scale. The data points are shown in grey for the cases where the pressure information was unavailable. The pressures at which membranes have been studied for CO_2_/CH_4_ separation range from 0.2 to 20 Bar. It is observed that larger pressures (red dots) were used for the materials located further below the Pineau and Robeson limits, and for membranes with lower permeability. The lower pressures (blue dots) were used for materials located close to the Pineau and Robeson limits.

**FIGURE 4:**
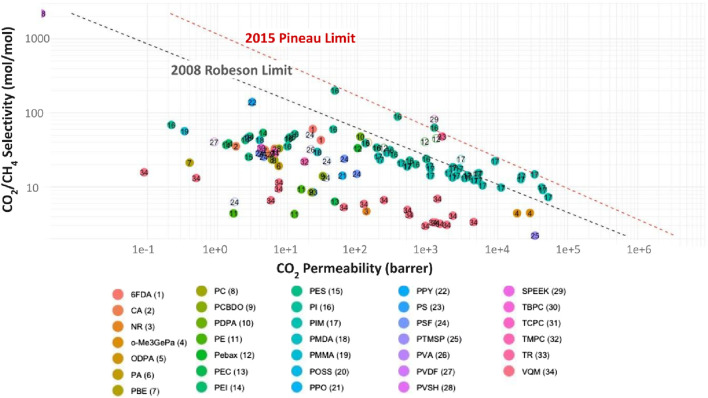
CO_2_/CH_4_ selectivity vs. CO_2_ permeability for different polymer membranes in gas separation, indicating the polymer materials with different numbers and colors guidelines. Based on (Park et al., 2008; Koros et al., 1988; Puleo et al., 1989; Sada et al., 1989; Mizumoto et al., 1993; Hachisuka et al., 1995; Pinnau, 1996; Al-Masri et al., 1999; Zimmerman and Koros, 1999; Lin and Chung, 2001; Yang et al., 2001; Nagel et al., 2002; Vu et al., 2003; BUDD et al., 2005; Shida et al., 2006; Wang et al., 2007; Ghanem et al., 2009; Fritsch et al., 2011; Ma et al., 2012; Sanders et al., 2013; Li et al., 2015; Shen et al., 2015; Xin et al., 2015; Althumayri et al., 2016; Ebrahimi et al., 2016; Bernardo et al., 2017; Castarlenas et al., 2017; Galizia et al., 2017; Alberto et al., 2018; Feijani et al., 2018; Li et al., 2018; Al-Maythalony, 2019; Comesaña-Gándara et al., 2019; Kheirtalab et al., 2020; Li et al., 2020; Raouf et al., 2020; Yang et al., 2020; Luque-Alled et al., 2021a; Luque-Alled et al., 2021b; Sainath et al., 2021; Zhao et al., 2021; Mohsenpour et al., 2022).

Figure 4 displays the same set of data, but in this case the polymer material is indicated with the color scale (Park et al., 2008; Koros et al., 1988; Puleo et al., 1989; Sada et al., 1989; Mizumoto et al., 1993; Hachisuka et al., 1995; Pinnau, 1996; Al-Masri et al., 1999; Zimmerman and Koros, 1999; Lin and Chung, 2001; Yang et al., 2001; Nagel et al., 2002; Vu et al., 2003; BUDD et al., 2005; Shida et al., 2006; Wang et al., 2007; Ghanem et al., 2009; Fritsch et al., 2011; Ma et al., 2012; Sanders et al., 2013; Li et al., 2015; Shen et al., 2015; Xin et al., 2015; Althumayri et al., 2016; Ebrahimi et al., 2016; Bernardo et al., 2017; Castarlenas et al., 2017; Galizia et al., 2017; Alberto et al., 2018; Feijani et al., 2018; Li et al., 2018; Al-Maythalony, 2019; Comesaña-Gándara et al., 2019; Kheirtalab et al., 2020; Li et al., 2020; Raouf et al., 2020; Yang et al., 2020; Luque-Alled et al., 2021a; Sainath et al., 2021; Zhao et al., 2021; Mohsenpour et al., 2022). The trends discussed above can be correlated to polymer chemistry by analyzing the clusters of points on the graph. The use of conventional polymers, such as CA, PC, and PMDA, require higher operating pressures, concentrating below the Robeson limit. The recently developed polymers are between the strip formed by the limits of Robeson and Pineau on the highest permeability values. Between these limits, two groups of polymers can be discerned (Atelge et al., 2021): TRs with higher selectivity, lower permeabilities, and noticeable presence of PI, located at the left end of the band,; and (Occupational Safety and Health Administration OSHA, 2019) the PIMs, which operate at pressures below 1 bar and are located at the far right of the range, with lower selectivity and higher permeability. These results indicate that progress in MMMs that can operate at lower pressure for medium and small biogas systems may be possible with TRs and PIMs, due to their higher free volume and higher selectivity and permeability values. The use of TR and PIMs discards the need for high pressure in which plasticization and aging increase membrane failure rates. The polymers at the right side of the graph, showing higher values of CO_2_ permeability are both the PIM-1 and the PTMSP, however, the CH_4_/CO_2_ selectivity of PIM-1 is around 7 times higher than PTMSP (16.2 for PIM-1 vs 2.2 for PTMSP).

### 3.2 Analysis of fillers in mixed-matrix-membranes using PIM-1 for CH_4_/CO_2_ separation

**FIGURE 5:**
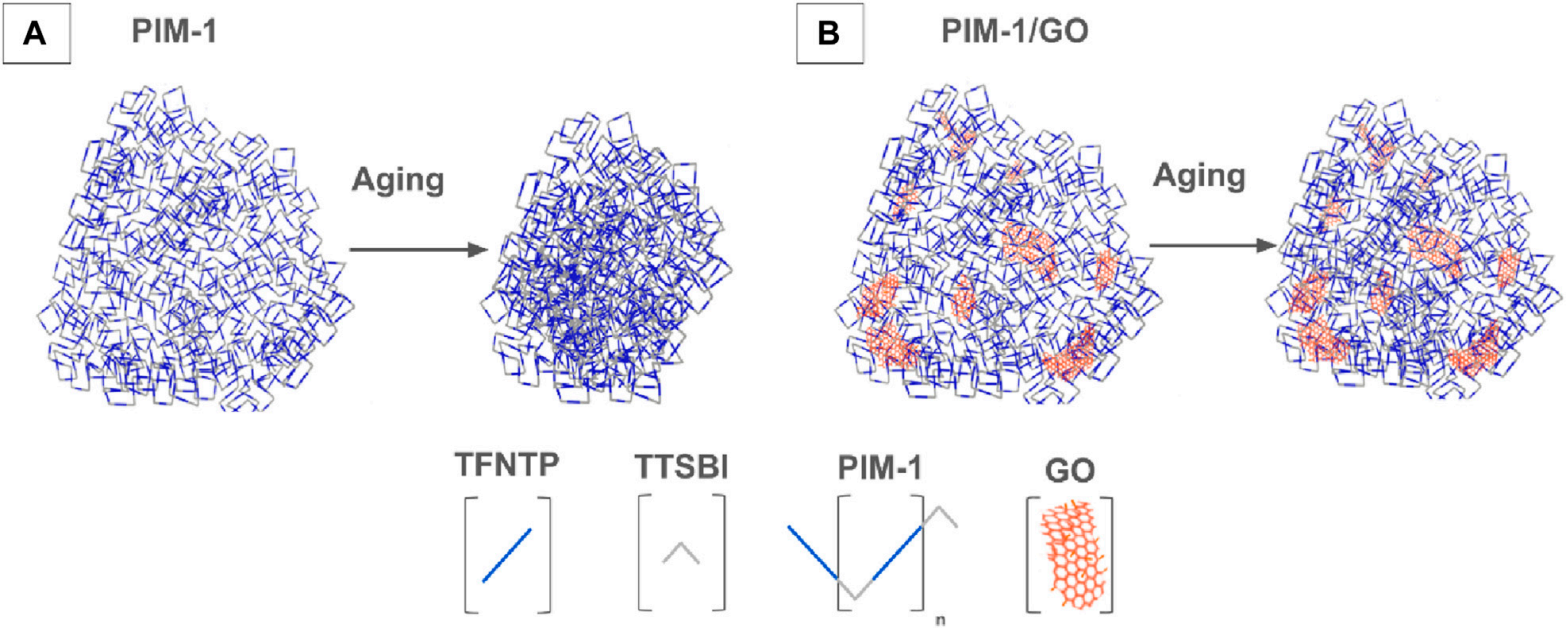
Illustration of **(A)** the aging mechanism in PIM-1 MMM and **(B)** the stability enhancement when using GO as filler. Based on (Bernardo et al., 2017; Alberto et al., 2018).

The current membranes technology for gas separation meets two major challenges: plasticization and aging, which are more predominant at higher pressures (Baena-Moreno et al., 2019). In CH_4_/CO_2_ separation, CO_2_ interacts with the polymer chains of the membranes, generating swelling of the structure and plasticization. Plasticization causes greater flexibility of the chains, increasing the free volume in the structure and causing higher permeability values and lower selectivity values. It is a behavior that usually accelerates when the polymer is subjected to high gas pressures (Rowe et al., 2009), occurring regardless of membrane thickness but accelerating in thinner membranes (Víquez et al., 2018). Glassy polymers exhibit a characteristic behavior of, in which large proportions of free volume are a result of packing errors and chain mobility limitations (Ali et al., 2017). The chemical structure of a glassy polymer, like PIM, provides a high permeability; however, this structure is not in thermodynamic equilibrium conditions, so it tends to reorganize over time to reduce the free energy and approach equilibrium states, i.e., aging, which reduces free volumes and, consequently, permeability. Figure 5A illustrates the aging of PIM-1, in which the pores of the structure collapse with time and the free volume is reduced (Bernardo et al., 2017). The reduction of free volume affects the CO2 permeability through the membrane. As with plasticization, increasing the thickness reduces the advance of the aging phenomenon on the material (Alberto et al., 2018).

**FIGURE 6:**
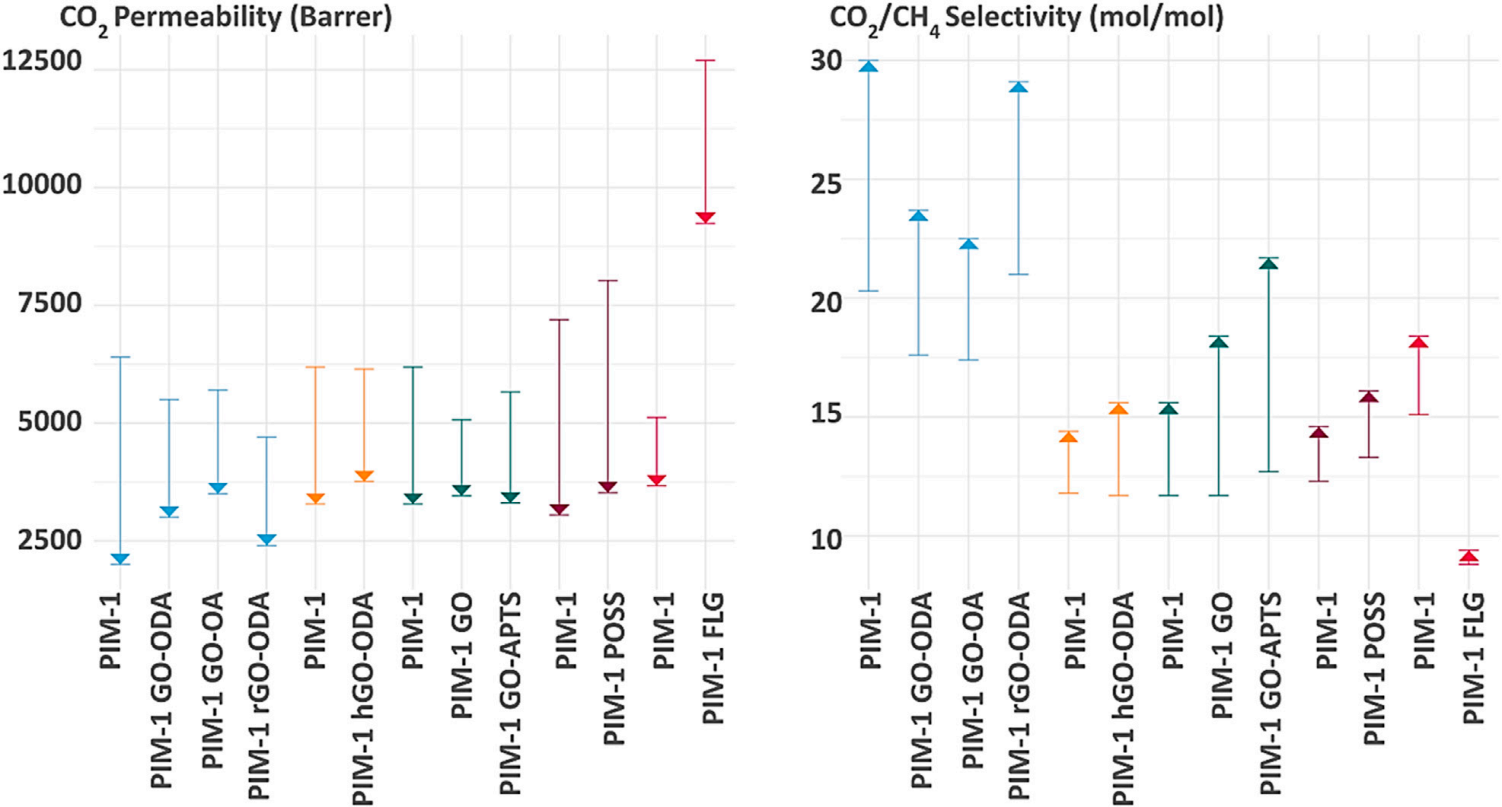
Effect of different fillers on permeability of CO_2_ (barrer) and CO_2_/CH_4_ selectivity (mol/mol) from 0 to 155 days in PIM-1 MMM. Based on (Althumayri et al., 2016; Alberto et al., 2018; Luque-Alled et al., 2021a; Luque-Alled et al., 2021b; Mohsenpour et al., 2022).

**FIGURE 7:**
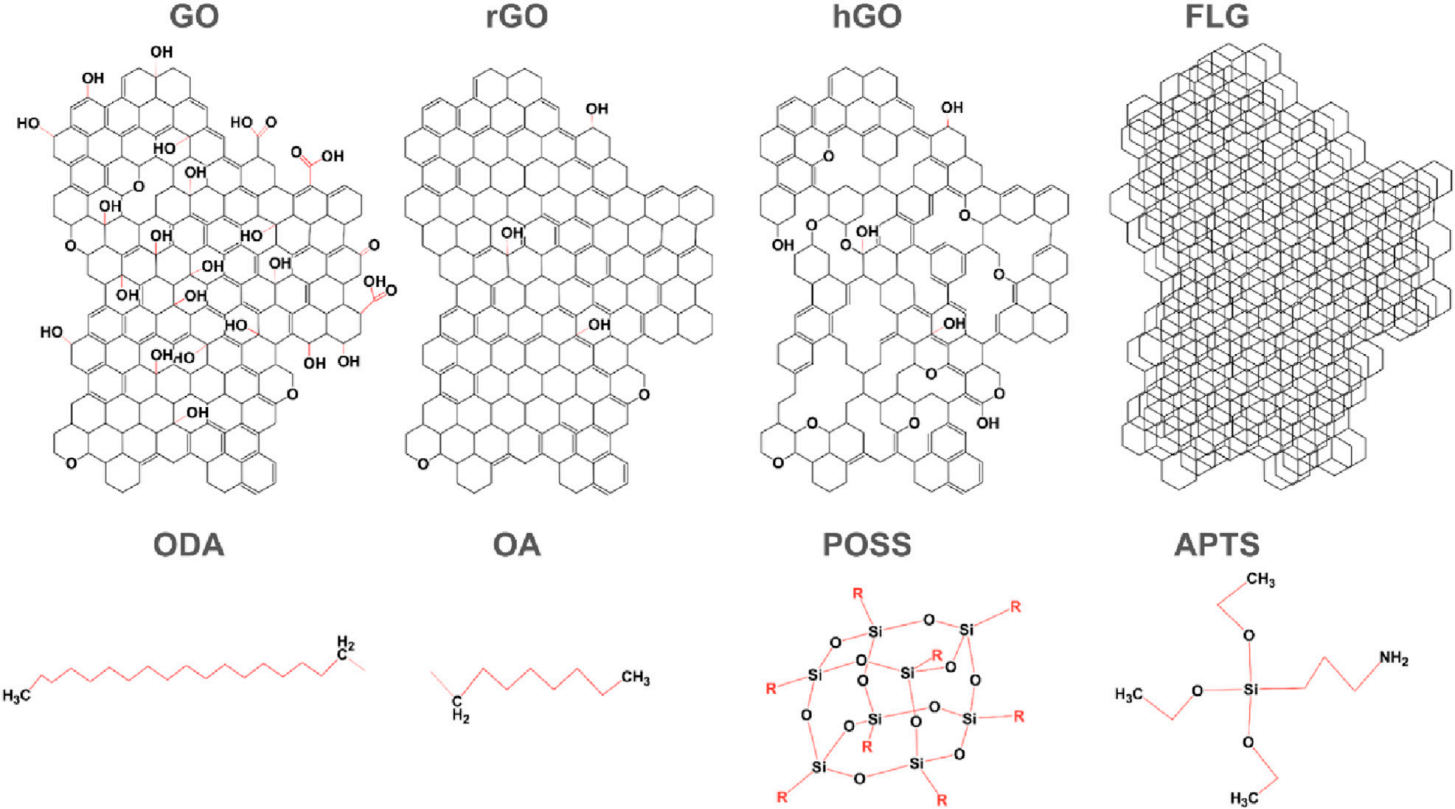
Illustration of chemical structures for different fillers in PIM-1 MMM. Based on (Althumayri et al., 2016; Alberto et al., 2018; Luque-Alled et al., 2021a; Luque-Alled et al., 2021b; Mohsenpour et al., 2022).

The incorporation of nanofillers in PIM-1 MMMs enhances the stability of the membrane over time, promising a transformative impact in the field of filtration technology. Amongst nanostructured carbon fillers and metal-organic nanostructures displays improved performance and lower cost compared to graphene nanoporous membranes (Baena-Moreno et al., 2019) synthesis of such polymer/nanocarbons composites can take place via different synthesis approaches *in situ* synthesis and mixing. Figure 5 illustrates the structural organization for PIM-1 and the interaction between GO. The GO acts as a mechanical enforcement that prevents the collapse or flow of the PIM-1 structure that causes aging and plasticization as illustrated in Figure 5B, The data of some MMMs were included as empty circles in Figure 4. The addition of fillers does not improve the gas separation performance in most cases, as the GO-MMMs do not concentrate at edges of the polymer clusters, do not exhibit any specific pattern, and do not display extreme values of selectivity or permeability. Incorporating fillers in MMM reduces the permeability slightly, by blocking the flow of molecules in the structure. The performance improvement over time of PIM-1 MMMs for different nanofillers can be observed in Figure 6, showing the behaviour of CO_2_ permeability drop over time and CO_2_/CH_4_ selectivity for eight different funcionalizations of PIM-1: GO (graphene oxide), GO-ODA (graphene oxide-octadecyl amine), rGO-ODA (reduced graphene oxide–octadecyl amine), rGO-OA (reduced graphene oxide–octyl amine), hGO-ODA (holey graphene oxide-octyl amine), GO-APTS and PIM-1 POSS (polyhedral oligomeric silsesquioxanes) Althumayri et al., 2016; Alberto et al., 2018; Luque-Alled et al., 2021a; Luque-Alled et al., 2021b; Mohsenpour et al., 2022). Molecular representations of every filler can be observed in Figure 7. The development of PIM/GO MMMs balances efficiency improvements with cost effectiveness, offering a viable path to widespread adoption of this filtration technologies (Chuah et al., 2021).

Significant improvements in delaying physical aging in PIM-1 membranes by incorporating modified graphene oxide (GO) (Alberto et al., 2018). Octylamine (OA) and octadecylamide (ODA) were used to branch GO, followed by a chemical reduction of graphene oxide. The optimal concentration for ODA-rGO, OA-GO, and OA-rGO was found to be 0.05%wt. According to Figure 6, over 160 days, CO_2_ permeability decreased less in modified membranes compared to pure PIM-1: 69% in pure PIM-1, 45% in PIM-1/0.05% GO-ODA, 49% in PIM-1/0.05% rGO-ODA, and 39% in PIM-1/0.05% wt rGO-OA. Comparisons indicate better performance of GO over rGO, and OA over ODA in terms of initial permeability and aging delay. Results for combinations of PIM-1 with OA and GO were not reported, leaving a gap in the analysis of these combined variables (Alberto et al., 2018).

According to (Luque-Alled et al., 2021a) they have modified graphene oxide to create “holey” graphene oxide (hGO), introducing voids within its structure that slightly improved the performance previously reported by (47), enhancing both the initial permeability and the result after 155 days. As it can be seen in Figure 6, PIM-1 hGO demonstrated an initial permeability of 6,146 barrer and a final permeability of 3,763 barrer, with an initial and final CO_2_/CH_4_ selectivity of 11.6 and 15.6 respectively. These results signify an appreciable advancement over non-holey graphene oxide. However, is notable that even the unmodified PIM-1 membrane in this study showed similar final CO_2_ permeabilities than some of the MMMs in the previous study, suggesting that the baseline PIM-1 membrane already exhibited excellent performance before functionalization (Luque-Alled et al., 2021a).

Further research by Luque-Alled et al. (2021b) explored different modifications of GO in PIM-1 membranes. In this study, they compared PIM-1, PIM-1 GO, and PIM-1 GO-APTS functionalization, resulting in improvements in selectivity rather than permeability after 150 days. The results, showed in the permeability and selectivity graph in Figure 6, showed a decline from initial permeability of 6,190 barrer for PIM-1 vs. 5,660 for PIM-1 GO-APTS and 5,072 for PIM-1 GO, and after 3 days the behavior was similar among all three materials, ranging from 3,283, 3,305 and 3,458 for PIM-1, PIM GO-APTS and PIM-1 GO, respectively. However, CO_2_/CH_4_ selectivity improvements were significant, with PIM-1 GO-APTS showing a rise from 12.6 to 21.7. These results suggest that APTS’s addition to GO not only enhances the distribution within the polymer matrix and contributes to a higher free volume, but also promoting more efficient CO_2_ and CH_4_ separation, playing a significant role in the transport of these two molecules (Luque-Alled et al., 2021a).

PIM-1 membranes were first functionalized with POSS (polyhedral oligomeric silsesquioxane) by (Rowe et al., 2009), marking an enhancement in gas separation performance more than a reduction in permeability reduction over time. This modification led to an enhancement of CO2 permeability of initial 8,026 barrers vs. 7,195 barrer of PIM-1. Both the initial and final permeabilities showed improvements, with final permeability values recorded at 3,524 barrer for PIM-1 POSS and 3,048 for PIM-1. Additionally, this modification resulted also in increases in selectivity, from initial values of 12.3 vs. 13.3 to finals 14.6 vs. 16.1, reflecting the benefits of POSS’s organosilicon structure, being remarkable as a functionalization that enhances both selectivity and permeability at the same time, a non-usual behavior in the rest of the previous functionalizations showed in Figures 6, 7, which follows the usual trade-off relationship between selectivity and permeability (Luque-Alled et al., 2021a).

When Few Layer Graphene (FLG) were added to the PIM-1, PIM-1 FLG in Figure 6, a notable enhancement in CO2 permeability was reported (Althumayri et al., 2016). Initially, permeability was reported to reach up to 12,700 Barrer, 248% higher than the 5,120 Barrer for the unmodified PIM-1. Even after aging, FLG-modified membranes maintained a permeability of 9,240 Barrer, compared to only 3,670 Barrer in the unmodified samples this also represents an enhancement in aging resistance, reporting a reduction of 27.24% for PIM-1 FLG vs. a 28.32% in PIM-1. This substantial increase highlights the effectiveness of FLG in improving the gas separation properties of PIM-1 membranes (Althumayri et al., 2016).

## 4 Conclusions and future perspective

This review comprehensively examined the historical progression in polymeric membranes and their approach to be incorporated in medium-scale biogas refining using mixed-matrix membranes. Throughout the 1980s and 1990s, the development and application of new polymers like PSF and the CA evolved, and these materials have now captured between 80% and 90% of the current CH_4_/CO_2_ separation market. The turn of the millennium saw the emerging of PIMs and TRs, two families of polymers with enhanced free volume and improved performance. Advancements in MMMs in the last decade, particularly with integrating carbonous “graphene like” materials, organosilicon materials, and MOFs, stand out in this journey. These recent innovations in materials present significant advantages over traditional refining methods, including enhanced selectivity and permeability, low-pressure operation, and improved resistance to plasticization and aging. This approach not only boosts operational efficiency but also enables the deployment of biogas technologies on smaller and medium scales, paving new paths for energy sustainability.

The polymers that stand out for their high permeability to CO_2_ and that enable lower operating pressures due to their structure and spatial organization, are PIM-1 and PTMSP. However, PIM-1 exhibits a more efficient CO_2_/CH_4_ selectivity that exceeds the PTMSP average by more than seven times (ratios of 16.2 for PIM-1 versus 2.2 PTMSP). Selectivity tests for the PIM-1 were carried out at pressures below 1 bar, with successful operational measurements even achieved at 0.2 bar. This pressure range favors the possibility of implementation in smaller-scale biogas systems compared to other membrane materials, indicating the most significant potential across different materials for anaerobic digestion at medium and minor scales.

The most recent studies show that incorporating nanofillers into polymeric membranes aims to improve their long-term performance, despite not doing so immediately, and may even slightly reduce it due to the use of bulky materials, creating physical obstruction of mass transport within the polymeric matrix. However, MMMs show increased efficiency throughout their useful life, strengthening the membrane resistance to plasticization and aging due to improved mechanical stability. The diversity of the results obtained outcomes highlight the complexity of membrane enhancements, reporting aging mitigation at the expense of reduced initial and final permeabilities, indicating a trade-off between longevity and performance. Conversely, some investigations showcase improved CO2/CH4 selectivity or initial and final permeabilities without a corresponding aging resistance, suggesting variability in nanofiller effects. Notably, few-layer graphene has shown outstanding increases in permeability, up to 300%. However, these advancements are not uniformly observed across all studies, with some polymers exhibiting superior performance without nanofillers.

Future research should focus on optimizing nanofiller dispersion and membrane fabrication processes to harness potential enhancements in aging resistance, permeability, and selectivity. The integration of graphene-like materials with other structurally stable nanofillers could further improve material stability and performance over time. Developing and refining membrane fabrication protocols is critical, as studies show substantial discrepancies in performance, even when comparing between PIM-1 reference material across the studies, with selectivity variations over 10 points and initial permeability differences exceeding 2,500 Barrer, causing differences over 30% between reference materials. Improved protocols for nanofiller dispersion could enhance initial material properties, leading to better overall membrane performance, combining aging resistance with improved initial and final permeability and selectivity.

To further enhance the development of mixed-matrix membranes, exploring materials or structural functionalizations that combine graphene-like materials with other nanofillers with high free volume, structural stability and high number of reaction points can be beneficial. Such combinations could create membranes with increased free volume and structural stability, potentially leading to significant improvements in both selectivity and permeability over time. While recent studies have shown enhancements in these properties individually, standardized protocols and optimized synthesis processes could improve results in baseline material and then integrate these advances more effectively, leading to reach superior membrane performance.
